# Supplementary material for: Knowledge and Practices toward Tuberculosis Case Identification among Accredited Drug Dispensing Outlets Dispensers in Magu District, Northwestern Tanzania
Source: Healthcare (Basel). 2024 Jan 10;12(2):168. doi: 10.3390/healthcare12020168 (PMC10815611; doi:10.3390/healthcare12020168)
Supplement: Supplementary file 1 [file healthcare-12-00168-s001.zip › healthcare-2725275-supplementary.pdf]

Questionnaire (English version)

Part I. Socio-demographic characteristics of the respondents

1) ID number of respondent__________________________________
2) ward ___________________
3) Village/Street_________________________________
4) Sex:
    1. Male
    2. Female
5) Age (Year): ____________
6) Ethnicity: _________________________________
7) Religion
    1. Muslim
    2. Christianity
    3. other specify__________________________________
8) Marital status
    1. Married
    2. single
    3. divorced
    4. widowed
9) Educational status:
    1. Primary education
    2. Secondary education
    3. College

4. University
5. other (specify) ____________________________

10) For how long have been working as an ADDO dispenser? _____________________

11) Do you migrate in from this place going to other places to search for the customers/employment?
    1. Yes
    2. No

12) Is there any nearby health facility around this area?
    1. Yes
    2. No

13) How long does it take to reach to the nearby health facility?
    1. 1-15 mins
    2. 16-30 mins
    3. 30 mins-1hr
    4. More than 1 hr.

Part II. Assessments of knowledge about PTB

1) Have you ever heard about the disease called TB?
    1. Yes
    2. No

2) If yes, from whom mainly?
    1. From friend (s)
    2. From health workers /Health extension workers
    3. From media (radio, TV poster etc.)

4. from patient
5. family member/friends
6. via reading books
7. via seminar/conferences
8. Other (specify): ___________________________

3) What do you think is the cause of PTB? (Check all that are mentioned)

1. Bacteria/germ
2. Virus
3. Fungus
4. witchcraft
5. shortage of food
6. unventilated house
7. Sexual overindulgence
8. hard work
9. poverty
10. living together with untreated TB patient
11. Cold air
12. Don't know
13. others (specify):______________________

4) What are some of the common symptoms of TB? (Check all that are mentioned)

1. Cough
2. Cough for 2 or more weeks
3. Cough up blood

4. Weight loss
5. Shortness of breath
6. Fever/sweat at night
7. Chest pain
8. Weakness or loss of appetite
9. Rash
10. Do not know
11. Other (specify)_____________

5) Do you know that TB can be transmitted from one person to another?
   1. Yes
   2. No

6) If yes to Q5 above, how can TB be transmitted from person to person? (Check all that are mentioned)
   1. Through droplets from coughing or sneezing of a person having TB
   2. Hand shaking of a person having TB
   3. Kissing TB infected person
   4. Drinking raw milk
   5. Eating TB infected meat
   6. Exposure to cold air
   7. Sharing cup with a person having TB
   8. Sharing utensils
   9. Chewing chat together
   10. Others (specify) :______________

7) Who do you think can transmit TB? (Check all that are mentioned)

1. A person who has active TB and not taking TB drugs
2. Anyone who has cough
3. A person who is diagnosed having TB and finished treatment
4. Others specify

8) Do you think that the transmission of PTB can be prevented?

1. Yes
2. No

9) If yes to Q8, How can TB be prevented (Check all that are mentioned)

1. Using separate room
2. Covering mouth and nose while coughing or sneezing
3. Avoiding sharing cups
4. Avoiding eating raw meat
5. Avoiding drinking raw milk
6. Through good nutrition
7. Others (specify):___________________

10) In your opinion, who can be infected with TB? (Check all that are mentioned)

1. Anybody
2. Poor people
3. Homeless people
4. Only alcoholics
5. Only people living with HIV
6. Others (specify): __________

11) Can TB be cured?

1. Yes
2. No

12) If Yes to Q11, How can someone with TB be cured? (Check all that are mentioned)

1. Traditional Medicine
2. Home Rest Without Medicine
3. Praying
4. Specific Drugs Given By Health Facility
5. Dots
6. Do Not Know
7. Others (Specify): _____________

13) Do you know the drugs for TB?

1. Yes
2. No
3. I don't know

Part III: presumptive TB case identification and referral practice of ADDOs dispensers

1) What is the average number of customers do you attend per day? _____________

2) Do you keep records of the signs and symptoms of the customers you attend?

    1. Yes
    2. No

3) What other services apart from dispensing drugs, do you offer to your clients?

    1. Counselling
    2. Providing health education

3. Referring to nearby health facility
4. Making follow-up after I attend them
5. All of the above
6. None of the above, mention……………………….

4) Have you ever seen person(s) with signs and symptoms of TB at your shop in the last one year?
    1. Yes
    2. No
5) Have you ever attended a person with signs and symptoms of TB in the last one year?
    1. Yes
    2. No.
6) What signs and symptoms did you consider to suspect that your customer was a TB case? Tick all that may apply
    1. Cough
    2. Cough for 2 or more weeks
    3. Cough up blood
    4. Weight loss
    5. Shortness of breath
    6. Fever/sweat at night
    7. Chest pain
    8. Weakness or loss of appetite
    9. Rash
    10. Do not know

11. Other (specify)_____________

7) What service did you offer to a TB case identified?

   1. Provided anti-TB drugs
   2. Referred to the nearby health facility
   3. Sent him/her to the traditional/ witch doctors
   4. Provided health education on TB and the client left
   5. Provided anti-TB drugs and health education
   6. Provide anti-TB drugs and referred to nearby health facility
   7. Didn’t do anything
   8. Multiple responses, indicate………………..or add responses……………………..
   ……………………………………………………………………………………

8) Do you have a tendency of referring persons with symptoms of TB/seriously sick to advanced treatment?

   1. Yes
   2. No

9) If yes, where do you refer to advanced treatment?

   1. Nearby health facility
   2. Other specialists (traditional healers)

10) If you have ever referred a patient to any of the mention category above, how many people with presumptive TB symptoms did you refer? Please specify: ____________________

11) Have you heard back from the patients you referred after their visit to health facilities?

   1. Yes
   2. No

12) If yes, what was their status after visiting healthcare facility?**Indicate numbers**

1. Non TB cases: ______________
2. Sputum examined and confirmed TB cases: _________
3. Treatment completed: _________________
4. On treatment: ______________
5. Lost to follow up: ______________
6. Other, Please Specify:________________

Part IV: Factors affecting TB case identification and referral practice of ADDOs dispensers

1) Have you ever attended any TB training?
    1. Yes
    2. No
2) Are you supplied with national TB guideline and other supplements?
    1. Yes
    2. No
3) Do you face any challenge in identifying the presumptive TB case?
    1. Yes
    2. No
4) What challenges do meet in identifying the presumptive TB case? Tick all that may apply
    1. Lack of knowledge on TB in general
    2. Lack of guideline in managing TB
    3. Poor presentation of sign and symptoms by the customer
    4. Inadequate skills in history taking and physical examination
    5. Lack of own laboratory equipment for diagnosis of TB

6. Family and Patient's interests
7. Others, mention _________________

5) What challenges do you face while referring a person with symptoms of TB to advanced treatment? Tick all that may apply

1. Lack of common organized referral system with the health facilities
2. Ill condition of the client
3. Mistrust of ADDO dispensers practice by health practitioners
4. Poor organizational and other structural systems of the government towards and the ADDO dispensers andhealth system
5. Family and patients interests
6. Fear to loose trust by our customers
7. Inadequate skills in referring clients
8. Other, please mention_______________

<u>THANK YOU FOR YOUR PARTICIPATION</u>

Questionnaire (Swahili version)

Sehemu ya I. Sifa za kijamii na kidemografia za wahojiwa

1) Nambari ya kitambulisho cha mhojiwa………………..

2) Kata ______________________________

3) Kijiji/Mtaa______________________________

4) jinsia

1. Mwanaume
2. Mwanamke

5) Umri (Miaka**):** __________

6) Kabila: ______________________________

7) Dini

1. Muislamu
2. Ukristo
3. bainisha mengine______________________________

8) Hali ya ndoa

1. Kuolewa
2. single
3. talaka
4. mjane

9) Hali ya elimu:

1. Elimu ya msingi
2. Elimu ya sekondari
3. Chuo

4. Chuo kikuu
5. nyingine (taja) ______________________________________

10) Ni kwa muda gani umekuwa ukifanya kazi kama muuza DLDM? ____________________

11) Je, unahama kutoka eneo hili kwenda sehemu nyingine kutafuta wateja/ajira?

1. Ndiyo
2. Hapana

12) Je, kuna kituo chochote cha afya karibu na eneo hili?

1. Ndiyo
2. Hapana

13) Inachukua muda gani kufika kwenye kituo cha afya kilicho karibu?

1. Dakika 1-15
2. Dakika 16-30
3. Dakika 30-saa 1
4. Zaidi ya saa 1.

Sehemu ya II. Tathmini ya maarifa kuhusu PTB

1) Je, umewahi kusikia kuhusu ugonjwa unaoitwa TB?

1. Ndiyo
2. Hapana

2) Kama ndiyo, kutoka kwa nani hasa?

1. Kutoka kwa rafiki
2. Kutoka kwa wahudumu wa afya/wahudumu wa afya
3. Kutoka kwa vyombo vya habari (redio, bango la TV n.k.)
4. Kutoka kwa mgonjwa

5. Wanafamilia/marafiki
6. Kupitia kusoma vitabu
7. Kupitia semina/kongamano
8. Nyingine (taja): ___________________________________

3) Unafikiri nini sababu ya kifua kikuu? (Angalia yote yaliyotajwa)

1. Bakteria/kiini
2. Virusi
3. Kuvu
4. Uchawi
5. Upungufu wa chakula
6. Nyumba isiyo na hewa
7. Kujiingiza katika ngono kupita kiasi
8. Kazi ngumu
9. Umaskini
10. Kuishi pamoja na mgonjwa wa TB ambaye hajatibiwa
11. Hewa baridi
12. Sijui
13. Sababu nyinginezo (taja):______________________________

4) Je! ni baadhi ya dalili za kawaida za TB? (Angalia yote yaliyotajwa)

1. Kikohozi
2. Kohoa kwa wiki 2 au zaidi
3. Kohoa hadi damu
4. Kupungua uzito

5. Kukosa pumzi
6. Homa/jasho usiku
7. Maumivu ya kifua
8. Udhaifu au kupoteza hamu ya kula
9. Upele
10. Sijui
11. Nyingine (taja) _____________

5) Je, unajua kwamba TB inaweza kuambukizwa?

1. Ndiyo
2. Hapana

6) Kama ndiyo kwa Q5 hapo juu, TB inawezaje kuambukizwa kutoka kwa mtu mmoja hadi mwingine? (Angalia yote yaliyotajwa)

1. Kupitia matone yanayotokana na kukohoa au kupiga chafya ya mtu mwenye TB
2. Kushikana mikono kwa mtu mwenye TB
3. Kumbusu mtu aliyeambukizwa TB
4. Kunywa maziwa mabichi
5. Kula nyama yenye TB
6. Mfiduo wa hewa baridi
7. Kushiriki kikombe na mtu mwenye TB
8. Vyombo vya kugawana
9. Gumzo la kutafuna pamoja
10. Nyingine (taja) :___________

7) Unafikiri ni nani anaweza kuambukiza TB? (Angalia yote yaliyotajwa)

1. Mtu ambaye ana TB hai na asiyetumia dawa za TB
2. Mtu yeyote ambaye ana kikohozi
3. Mtu anayegundulika kuwa ana TB na kumaliza matibabu
4. Wengine hutaja

8) Je, unafikiri kwamba maambukizi ya PTB yanaweza kuzuiwa?

1. Ndiyo
2. Hapana

9) Ikiwa Ndiyo kwa swali la 8, TB inawezaje kuzuiwa (Angalia yote yaliyotajwa)

1. Kutumia chumba tofauti
2. Kufunika mdomo na pua wakati wa kukohoa au kupiga chafya
3. Kuepuka kugawana vikombe
4. Kuepuka kula nyama mbichi
5. Kuepuka kunywa maziwa mabichi
6. Kupitia lishe bora
7. Nyingine (taja):______________________________

10) Kwa maoni yako, ni nani anaweza kuambukizwa TB? (Angalia yote yaliyotajwa)

1. Mtu yeyote
2. Watu maskini
3. Watu wasio na makazi
4. Walevi tu
5. Watu wanaoishi na VVU pekee
6. Nyingine (taja): __________

11) Je, TB inaweza kuponywa?

1. Ndiyo
2. Hapana

12) Kama Ndiyo kwa Swali la 11, Je, mtu mwenye TB anawezaje kuponywa? (Angalia yote yaliyotajwa)

1. Tiba Asilia
2. Kupumzika Nyumbani Bila Dawa
3. Kuomba
4. Dawa Maalum Zinazotolewa na Kituo cha Afya
5. Dots
6. Sijui
7. Nyingine (Taja): ___________

13) Je, unazijua dawa za TB?

1. Ndiyo
2. Hapana
3. Sijui

Sehemu ya Tatu: utambuzi wa kesi ya TB dhahania na mazoezi ya rufaa ya watoa dawa za DLDM

1) Je, ni wastani wa idadi gani ya wateja unaohudhuria kwa siku? __________

2) Je, unaweka kumbukumbu za ishara na dalili za wateja unaohudhuria?

1. Ndiyo
2. Hapana

3) Je, ni huduma gani nyingine mbali na kusambaza dawa unazotoa kwa wateja wako?

1. Ushauri
2. Kutoa elimu ya afya
3. Kurejelea kituo cha afya kilicho karibu
4. Kufanya ufuatiliaji baada ya kuhudhuria
5. Yote hapo juu
6. Hakuna kati ya zilizo hapo juu, taja………………………….

4) Je, umewahi kuona watu wenye dalili za TB katika mwaka mmoja uliopita?

1. Ndiyo
2. Hapana

5) Je, umewahi kuhudumia mtu mwenye dalili za TB katika mwaka mmoja uliopita?

1. Ndiyo
2. Hapana

6) Je, ni dalili gani ulizingatia kushuku kuwa mteja wako alikuwa na TB? Weka alama kwenye yote yanayoweza kutumika

1. Kikohozi
2. Kikohozi kwa wiki 2 au zaidi
3. Kohoa hadi damu
4. Kupunguza uzito
5. Kukosa pumzi
6. Homa/jasho usiku
7. Maumivu ya kifua
8. Udhaifu au kupoteza hamu ya kula
9. Upele

10. Sijui

11. Nyingine (taja) _____________

7) Ulitoa huduma gani kwa mgonjwa wa TB aliyetambuliwa?

1. Kutolewa kwa dawa za kuzuia TB
2. Kupelekwa kwenye kituo cha afya kilicho karibu
3. Kumpeleka kwa waganga wa kienyeji/wachawi
4. Kutolewa kwa elimu ya afya
3. Kurejelea kituo cha afya kilicho karibu
4. Kufanya ufuatiliaji baada ya kuhudhuria
5. Yote hapo juu
6. Hakuna kati ya zilizo hapo juu, taja………………………….

8) Ulitoa huduma gani kwa mgonjwa wa TB aliyetambuliwa?

1. Kutolewa kwa dawa za kuzuia TB
2. Kupelekwa kwenye kituo cha afya kilicho karibu
3. Kumpeleka kwa waganga wa kienyeji/wachawi
4. Kutoa elimu ya afya juu ya TB na mteja kuondoka
5. Kutoa dawa za kuzuia TB na elimu ya afya
6. Kutoa dawa za kuzuia TB na rufaa kwenye kituo cha afya kilicho karibu
7. Hakufanya chochote
8. Majibu mengi, onyesha…………………..au ongeza majibu………………………..

………………………………………………………………………………………

9) Je, una tabia ya kuwaelekeza watu wenye dalili za TB/ wagonjwa mahututi kwenda matibabu ya juu?

1. Ndiyo
2. Hapana

10) Ikiwa ndio, unawatuma wapi kwa ajili ya matibabu ya hali ya juu?

1. Kituo cha afya kilicho karibu
2. Wataalamu wengine (waganga wa kienyeji)

11) Iwapo umewahi kuelekeza mgonjwa wa aina yoyote kati ya zilizotajwa hapo juu, ni watu wangapi waliokuwa na dalili za TB uliwaelekeza? Tafadhali taja: ____________________

12) Je, umesikia maoni kutoka kwa wagonjwa uliowapa rufaa baada yawao kutembelea kwenye vituo vya afya?

1. Ndiyo
2. Hapana

13) Kama ndiyo, hali yao ilikuwaje baada ya kutembelea kituo cha huduma ya afya? Onyesha nambari

1. Watu wasio na TB: ______________
2. Makohozi yaliyochunguzwa na kuthibitishwa visa vya TB: _________
3. Matibabu yamekamilika: _______________
4. Kwenye matibabu: ______________
5. Imepotea kufuatilia: ______________
6.
7. Nyingine, Tafadhali Taja:________________

Sehemu ya IV: Mambo yanayoathiri utambuzi wa kesi za TB na mazoezi ya rufaa ya watoa dawaza DLDM

1) Je, umewahi kuhudhuria mafunzo yoyote ya TB?

1. Ndiyo
2. Hapana

2) Je, umepewa mwongozo wa kitaifa wa TB na virutubisho vingine?

1. Ndiyo
2. Hapana

3) Je, unakabiliwa na changamoto yoyote katika kutambua kisa cha TB?

1. Ndiyo
2. Hapana

4) Ni changamoto gani hukutana nazo katika kutambua kisa cha TB? Weka alama kwenye yote yanayoweza kutumika

1. Ukosefu wa elimu juu ya TB kwa ujumla
2. Ukosefu wa mwongozo katika kudhibiti TB
3. Uwasilishaji mbaya wa ishara na dalili na mteja
4. Ujuzi usiofaa katika kuchukua historia na uchunguzi wa kimwili
5. Ukosefu wa vifaa vya maabara vya kutambua TB
6. Maslahi ya Familia na Mgonjwa
7. Nyingine, taja ______________________________

5) Je, ni changamoto gani unakumbana nazo unapompeleka mtu mwenye dalili za TB kwenye matibabu ya hali ya juu? Weka alama kwenye yote yanayoweza kutumika

1. Ukosefu wa mfumo wa rufaa uliopangwa pamoja na vituo vya afya
2. Hali mbaya ya mteja
3. Kutokuamini kwa watoa dawa za DLDM kunafanywa na wahudumu wa afya
4. Mifumo mbovu ya kimuundo na kimuundo ya serikali kuelekea wasambazaji wa DLDM na mfumo wa afya.
5. Maslahi ya familia na wagonjwa
6. Hofu ya kupoteza uaminifu kwa wateja wetu
7. Ujuzi duni katika kuwaelekeza wateja
8. Nyingine, tafadhali taja________________________________

ASANTE KWA USHIRIKI WAKO
